# Crisis due to war: anxiety, depression and stress in the population of 13 Latin American countries

**DOI:** 10.3389/fpsyt.2023.1218298

**Published:** 2023-07-20

**Authors:** Christian R. Mejia, Aldo Alvarez-Risco, Scherlli Chamorro-Espinoza, Jorge Andrés Castillón-Lozano, Medally C. Paucar, Valeria J. Padilla-F, José Armada, Martin A. Vilela-Estrada, Victor Serna-Alarcón, Shyla Del-Aguila-Arcentales, Jaime A. Yáñez

**Affiliations:** ^1^Universidad de Huánuco, Huáuco, Peru; ^2^Universidad Tecnológica del Perú, Lima, Peru; ^3^Universidad de Aquino Bolivia, Santa Cruz, Bolivia; ^4^Asociación Médica de Investigación y Servicios en Salud, Lima, Peru; ^5^Facultad de Medicina, Grupo de investigación Infettare, Universidad Cooperativa de Colombia, Medellín, Colombia; ^6^Asociación de Sociedades Científicas de Estudiantes de Medicina de Colombia (ASCEMCOL), Bogota, Colombia; ^7^Universidad Continental, Huancayo, Peru; ^8^Federación Latinoamericana de Sociedades Científicas de Estudiantes de Medicina, Asunción, Paraguay; ^9^Universidad Autónoma Juan Misael Saracho, Tarija, Bolivia; ^10^Escuela Profesional de Medicina Humana, Universidad Privada Antenor Orrego, Trujillo, Peru; ^11^Hospital José Cayetano Heredia, EsSalud, Piura, Peru; ^12^Escuela de Posgrado, Universidad San Ignacio de Loyola, Lima, Peru; ^13^Vicerrectorado de Investigación, Universidad Norbert Wiener, Lima, Peru

**Keywords:** health, mental health, war, anxiety, depression, stress, Latin

## Abstract

Sustainability may be at risk in a population that has altered health, according to Sustainable Development Goal 3 (SDG 3): Health and well-being. The ongoing conflict between Russia and Ukraine could jeopardize SDG 3, specifically the mental health of the population. The present study sought to determine the association between severe anxiety, depression and stress in population of 13 Latin American countries according to fear about the war conflict. It was a cross-sectional, analytical and multicenter study. Anxiety, depression and stress were measured with the DASS-21 test (Cronbach’s Alpha: 0.97) and fear due to an armed crisis with a questionnaire already validated in Latin America (Cronbach’s Alpha: 0.92), which was also adjusted for sex, age, education level and country of residence. Descriptive and analytical statistics were obtained. Of the 2,626 respondents, the main fear was that weapons of mass destruction would be used. In the multivariate models, strong associations were found between fear of a possible world-scale armed conflict and having severe or very severe levels of anxiety (aPR: 1.97; 95% CI: 1.64–2.36; value of *p* <0.001), depression (aPR: 1.91; 95% CI: 1.54–2.36; value of *p* <0.001) or stress (aPR: 2.05; 95% CI: 1.63–2.57; value of *p* <0.001). Sustainability linked to SDG 3, specifically mental health, is affected by this type of significant events, given the possible global war crisis that could trigger major events, even more so if added to the deterioration already experienced by COVID-19 in the Latin American region, insecurity and constant political uncertainty.

## Introduction

Sustainability needs to be ensured in the population for its harmonious life. To contribute to this sustainability, the Sustainable Development Goals (1) were proposed in 2015, which address the economic, environmental, and social aspects of society. SDG 3 aims to ensure a healthy life and promote well-being for all at all ages; specifically, target 3.4 commits to reducing by 2030, by one-third premature mortality from non-communicable diseases through prevention and treatment and promoting mental health and well-being (2). In 2015, mental health was included as one of the main development objectives in the search for greater sustainability, due to the fact that mental problems are affected by social determinants, intertwined with the concern, perceptions and consequences of armed conflicts. For this reason, all events that generate alterations in mental health will end up going against the sustainability of society. For this reason, all events such as witnessing a war conflict, can generate alterations in mental health that will end up going against the sustainability of society since it could even cause psychiatric disorders (3).

COVID-19 has already generated a negative impact on the mental health of the population (4–6), more specifically as depression (7–9), anxiety (10, 11) and stress (12–14) so any situation can worsen the mental health of the population. Just talking about a world war reminds us that there are weapons of mass destruction that are so powerful that they could leave their mark on the environment (15–17), soil (18–20), air and others for many centuries or even millennia (21). Similarly, during the last century the world experienced the Second World War when Hitler ordered the Nazi invasion of Poland (22).

Sustainability is a crucial concept that requires meeting present needs while safeguarding the capacity of future generations to meet their own needs. It’s a comprehensive approach that takes into account the environmental, social, and economic aspects of development. The United Nations established Sustainable Development Goal 3, which aims to promote healthy lives and well-being for all at all ages, with a particular focus on mental health. Mental health plays a pivotal role in overall well-being, contributing to sustainable development in various ways. Optimal mental health is fundamental to individual well-being, enabling people to enjoy a fulfilling life and contributing to better health and quality of life. Moreover, mental health has a significant impact on productivity and the economy, as mental health issues can negatively affect participation in society and work, resulting in decreased economic performance. Addressing mental health is crucial to promote social justice by ensuring equal access to health services and creating a more inclusive society. Lastly, promoting mental health and preventing mental disorders can reduce the burden of disease, thus leading to a healthier and more sustainable society.

In this context, the concern of the population reached a critical level in February 2022, when Russia invaded Ukrainian territory to start a war with this nation (23–25). The United States and Europe gave their support to Ukraine (26–28), as did China to its Russian ally (29–31). Fortunately, after a few months, the possible military conflict stabilized and did not escalate, however, at the time of the events it was thought that the worst could happen (32). It is important to mention that, at that time, the world was going at the same time of the COVID-19 pandemic (33, 34).

This already had some repercussions on the mental scale, and there were multiple multicenter reports of large populations that show that in this pandemic the mental sphere of the most affected populations deteriorated (35, 36). This also happened in Latin America, which was the most affected in terms of *per capita* mortality, and other countries that, due to their precarious health systems, suffered greatly and have still not fully recovered (37). This situation could have been worst but the high acceptance of COVID-19 vaccination in Latin America helped to partially alleviate the fear caused by the pandemic (38, 39). Therefore, as we are coming out of a pandemic and given the scarcity of research on the mental consequences in individuals from countries that were not directly involved in a war like the current one, it is important to analyze how the stress, anxiety and depression at this specific moment in recent history in Latin America. Knowing that although a pandemic was important in the incidence of mental health, having the possibility of a third world war in 2022 was a very important breaking point to have greater damage to the already broken mental health (40, 41).

Therefore, the hypothesis established that there was a difference between three of the mental pathologies most commonly affected according to the perception that a new large-scale war would be generated. The objective of this study was to determine whether there is an association between anxiety, depression and severe stress according to fear of a possible third world war in the context of the Russia-Ukraine conflict in 13 countries of Latin America.

## Materials and methods

### Study design and sample

Cross-sectional analytical research was conducted. Due to the suddenness of the conflict, it was necessary to use this design by applying a single survey during the initial weeks of the Russia-Ukraine conflict. The survey lasted from March 5 to April 30, 2022; that is, from the second to the ninth week since the war started. This was conducted in the countries of Colombia (*n* = 584), Paraguay (*n* = 406), Peru (*n* = 393), Bolivia (*n* = 331), Ecuador (*n* = 238), Mexico (*n* = 204), Panama (*n* = 175), Brazil (*n* = 130) and others (Argentina, Cuba, Guatemala, Chile, and Honduras; each with less than 80 surveys). In each of these countries, most respondents were young urban population, with technical or university studies, which is the most accessible population through virtuality and occupies a significant percentage of the population in each of the Latin American countries.

In the first phase of the research, the validation of the instrument measuring the perception of fear or worry about an imminent world war was carried out. A non-probabilistic sampling was carried out, since it was never intended to extrapolate the results to the entire population, but it was desired to have enough respondents to obtain statistical associations. Due to this, the statistical power of the main crosstabs was calculated, being adequate the power for the crosstabs of the variable perception of fear or worry about an imminent world war according to anxiety, depression and stress (statistical power of 100% for each crosstabs). Using this analysis, it was possible to be certain that the main crosstabs had the necessary number of respondents to be able to trust the crosstabs and statistical results, all based on excellent statistical power (80% is the minimum power that is required, but the closer to 100% the better). We included those who wished to participate in the study (47 did not), who were of legal age (22 were minors) and who resided in a Latin American country during the survey period (38 resided in other countries). Those who left the questionnaire blank (8 persons), those who gave inconsistent answers (2 persons), those who did not give answers to the DASS-21 test (253 persons), to the test of fear or concern about an imminent world war (343 persons) or to the other adjustment variables (65 persons) were excluded.

### Variables and instruments

The dependent variables were three (anxiety, depression and stress), which were evaluated using the DASS-21 test. The survey asks about what happened in the last week through seven questions for the measurement of each one. All had four possible answers: never, a little, quite a lot or a lot. To obtain the results, the sum of the corresponding questions for each outcome was made, and according to the score, five possible results were obtained: normal values, as well as probabilities of having the pathology in low, moderate, severe or very severe ranges. The analytical statistics were dichotomized into severe (grouping severe and very severe) and non-severe (moderate, low and normal values). Alpha DASS-21 was calculated at 0.97.

For the measurement of the main independent variable, the test measuring the perception of fear or concern about an imminent world war was validated in the first phase of the research with the response of 1,684 Latin American residents during the Russia-Ukraine conflict. The validation process included obtaining preliminary analysis (skewness, kurtosis and communalities), exploratory factor analysis (Kaiser-Meyer-Olkin and Bartlett’s test) and confirmatory analysis (with the RMR, GFI, CFI, TLI, and RMSEA indices), as well as Cronbach’s Alpha measurement (overall: 0.92; factor 1 = 0.98 and factor 2 = 0.88); it should be noted that all the values obtained in the validation of the test were satisfactory (citation test fear, even if it is the preprint). Therefore, the prior validation process ensured that the instrument that measured fear in the face of a possible third world war was understandable and adapted to the reality of the respondents. For the analytical statistics, we categorized according to the score obtained from the total sum, being considered as those who were afraid of World War III those who were in the upper tercile of the total sum of the scores, being compared versus those who were in the middle plus the lower tercile. Our research also obtained the value of Cronbach’s Alpha, being 0.92.

We also considered the variables of sex (male or female), age (in years completed), level of education (up to technical or university level), country of residence (Colombia, Paraguay, Peru, Bolivia, Ecuador, Mexico, Panama, Brazil and others), perception of fear, depression and stress related to the country of residence (and political situation at risk of the inhabiting country).

### Ethical issues

The project was initiated days before the confirmation of the war conflict, and was urgently submitted to the ethics committee, to which the importance of an expedited review was explained (due to the need for a quick survey during the first weeks of the event). The committee gave its approval (Institutional Research Ethics Committee of the Norbert Wiener University, file 1,648–2022), especially because the surveys would be anonymous, there would be no contact with the respondents, the right to respond partially or totally to the survey would be respected, the objective and other characteristics of the research would be mentioned in the initial part, as well as not addressing vulnerable populations.

### Management and analysis of data

Once approval was obtained, the survey was carried out, for which two platforms were generated in Google Forms (one in Spanish and the other in Portuguese), which were available during the entire period previously mentioned. After the survey was completed, the information was debugged and reviewed according to the selection criteria; all this was done in a Microsoft Excel spreadsheet (Windows 2019 version). The information was then transferred to the Stata program (version 16), where the statistical analysis was performed.

The frequencies and percentages of the categorical variables were obtained; this is where Table 1 was generated with the percentage responses for each question of the fear test. For the analysis of the quantitative variables, their normality was first evaluated with the Shapiro Wilk statistical test, and when it was determined that they had a non-normal behavior, they were described with the median and interquartile ranges. Then we went on to the construction of each of the bivariate and multivariate cross tables, where generalized linear models (Poisson family, log link function and models for robust variances) were used to obtain the crude prevalence ratios (RPc), adjusted prevalence ratios (RPa), 95% confidence intervals (95%CI95%) and value of ps. In addition, epidemiological criteria were considered when entering all the adjustment variables, even if they were not statistically significant, as in the case of educational level, especially due to the knowledge that this variable is important in the population evaluated through previous research (42–44). In the final model, 0.05 was considered as the cut-off point for statistical significance.

**Table 1:** Perception of fear or concern about an imminent world war in Latin America.

In the face of an imminent world war, I am afraid or worried about	Strongly disagree	Disagree	Indifferent	Agree	Strongly agree
I am afraid of losing my life because of a new world war	20%	19%	22%	29%	10%
Seeing news and stories about the possibility of a new world war on social networks makes me nervous or anxious	16%	19%	24%	32%	9%
That my heart races or skips a beat when I think about a new world war	27%	24%	29%	16%	4%
That the events that are currently taking place will lead to a new world war	18%	16%	21%	35%	10%
That atomic bombs will be dropped or used on us	18%	14%	15%	31%	22%
May biological weapons be dropped or used against us	18%	13%	15%	32%	22%
Weapons of mass destruction being dropped or used on us	18%	13%	15%	31%	23%

## Results

Of the 2,626 respondents, 61.1% (1605) were female, the median age was 23 years (interquartile range: 21–28 years) and 89.7% (2356) were college graduates. The main fear was that weapons of mass destruction would be used (23% strongly agreed and 31% agreed), followed by that biological weapons would be used (22% strongly agreed and 32% agreed) or atomic bombs (22% strongly agreed and 31% agreed) (Table 1).

In the crossover of severe or very severe anxiety according to fear of a possible world war, a strong association was found (aPR: 1.97; 95% CI: 1.64–2.36; value of *p*<0.001). Furthermore, there was more anxiety in Ecuador (aPR a: 1.71; 95% CI: 1.02–2.84; value of *p* = 0.041), but the level of anxiety was lower the older the person was (aPR: 0.98; 95% CI: 0.96–0.99; value of *p* = 0.001) and among men (aPR: 0.74; 95% CI: 0.60–0.90; value of *p* = 0.002); adjusted for educational level (Table 2).

**Table 2:** Bivariate analysis of the fear of a possible world war according to severe or very severe anxiety in Latin American population.

Variable	Severe anxiety or >		cPR (IC 95%) value of *p*	
	No *n* (%)	Yes *n* (%)	Bivariate	Multivariate
World War III				
I am not afraid	1,535 (89.2)	186 (10.8)	Comparison category	Comparison category
Yes, I am afraid	690 (76.2)	215 (23.8)	2.20 (1.84–2.62) <0.001	1.97 (1.64–2.36) <0.001
Country				
Others	148 (89.7)	17 (10.3)	Comparison category	Comparison category
Colombia	477 (81.7)	107 (18.3)	1.78 (1.10–2.88) 0.019	1.57 (0.98–2.52) 0.061
Paraguay	331 (81.5)	75 (18.5)	1.79 (1.09–2.94) 0.021	1.80 (1.11–2.93) 0.061
Peru	343 (87.3)	50 (12.7)	1.23 (0.73–2.08) 0.426	1.32 (0.79–2.21) 0.283
Bolivia	276 (83.4)	55 (16.6)	1.61 (0.97–2.69) 0.067	1.58 (0.95–2.61) 0.075
Ecuador	191 (80.2)	47 (19.8)	1.92 (1.14–3.21) 0.014	1.71 (1.02–2.84) 0.041
Mexico	172 (84.3)	32 (15.7)	1.52 (0.88–2.64) 0.135	1.47 (0.85–2.53) 0.163
Panama	162 (92.6)	13 (7.4)	0.72 (0.36–1.44) 0.353	0.69 (0.35–1.36) 0.280
Brazil	125 (96.1)	5 (3.9)	0.37 (0.14–0.99) 0.047	0.49 (0.19–1.29) 0.149
Age (years)*	24 (21–29)	22 (20–25)	0.97 (0.95–0.98) <0.001	0.98 (0.96–0.99) 0.001
Sex				
Woman	1,319 (82.2)	286 (17.8)	Comparison category	Comparison category
Man	906 (88.7)	115 (11.3)	0.63 (0.52–0.77) <0.001	0.74 (0.60–0.90) 0.002
Education				
Technician or <	235 (87.0)	35 (13.0)	Comparison category	Comparison category
University students	1990 (84.5)	366 (15.5)	1.20 (0.87–1.66) 0.272	1.07 (0.78–1.47) 0.667

Crude prevalence ratios (cPR), adjusted prevalence ratios (aPR), 95% confidence intervals (95% CI) and value of ps were obtained with generalized linear models (Poisson family, log link function and models for robust variances). *Variable taken as quantitative; median (interquartile range) is shown in the descriptive part. We also found a strong association between severe or very severe depression and fear of a possible world war (aPR: 1.91; 95% CI: 1.54–2.36; value of *p*<0.001).

In addition, there was more depression in Paraguay (aPR: 2.09; 95% CI: 1.17–3.75; value of *p* = 0.013), Bolivia (aPR: 1.94; 95% CI: 1.06–3.53; value of *p* = 0.030) and Ecuador (aPR: 2.11; 95% CI: 1.16–3.86; value of *p* = 0.015); but the level of depression was lower the older the age (aPR: 0.97; 95% CI: 0.95–0.98; value of *p*<0.001) and among men (aPR: 0.77; 95% CI: 0.62–0.97; value of *p* = 0.024); adjusted for educational level (Table 3).

**Table 3:** Bivariate analysis of the fear of a possible world war according to severe or very severe depression in Latin American population.

Variable	Severe depression or >		cPR (IC 95%) value of p	
	No *n* (%)	Yes *n* (%)	Bivariate	Multivariate
World War III				
I am not afraid	1,571 (91.3)	150 (8.7)	Comparison category	Comparison category
Yes, I am afraid	736 (81.3)	169 (18.7)	2.14 (1.74–2.63) <0.001	1.91 (1.54–2.36) <0.001
Country				
Others	153 (92.7)	12 (7.3)	Comparison category	Comparison category
Colombia	508 (87.0)	76 (13.0)	1.79 (0.99–3.21) 0.051	1.52 (0.85–2.71) 0.158
Paraguay	344 (84.7)	62 (15.3)	2.10 (1.16–3.79) 0.014	2.09 (1.17–3.75) 0.013
Peru	354 (90.1)	39 (9.9)	1.36 (0.73–2.54) 0.327	1.44 (0.78–2.66) 0.242
Bolivia	283 (85.5)	48 (14.5)	1.99 (1.09–3.65) 0.025	1.94 (1.06–3.53) 0.030
Ecuador	196 (82.4)	42 (17.6)	2.42 (1.32–4.47) 0.004	2.11 (1.16–3.86) 0.015
Mexico	180 (88.2)	24 (11.8)	1.62 (0.83–3.14) 0.154	1.56 (0.81–2.99) 0.183
Panama	164 (93.7)	11 (6.3)	0.86 (0.39–1.90) 0.718	0.80 (0.37–1.77) 0.585
Brazil	125 (96.1)	5 (3.9)	0.53 (0.19–1.46) 0.220	0.72 (0.26–1.98) 0.527
Age (years)*	24 (21–29)	22 (20–25)	0.96 (0.94–0.98) <0.001	0.97 (0.95–0.98) <0.001
Sex				
Woman	1,380 (86.0)	225 (14.0)	Comparison category	Comparison category
Man	927 (90.8)	94 (9.2)	0.66 (0.52–0.82) <0.001	0.77 (0.62–0.97) 0.024
Education				
Technician or <	238 (88.1)	32 (11.9)	Comparison category	Comparison category
University students	2069 (87.8)	287 (12.2)	1.03 (0.73–1.45) 0.875	0.91 (0.65–1.27) 0.589

Crude prevalence ratios (cPR), adjusted prevalence ratios (aPR), 95% confidence intervals (95% CI) and value of ps were obtained with generalized linear models (Poisson family, log link function and models for robust variances). *Variable taken as quantitative; median (interquartile range) is shown in the descriptive part.

Likewise, a strong association was found between severe or very severe stress and fear of a possible world war (aPR: 2.05; 95% CI: 1.63–2.57; value of *p*<0.001). In addition, there was more stress in Colombia (aPR: 2.10; 95% CI: 1.08–4.07; value of *p* = 0.028), Paraguay (aPR: 2.55; 95% CI: 1.30–4.99; value of *p* = 0.007) and Ecuador (aPR: 2.53; 95% CI: 1.26–5.05; value of *p* = 0.009); and lower level of stress at older age (aPR: 0.97; 95% CI: 0.95–0.98; value of *p* = 0.001) and among men (aPR: 0.61; 95% CI: 0.47–0.79; value of *p*<0.001); adjusted for educational level (Table 4).

**Table 4:** Bivariate analysis of the fear of a possible world war according to severe or very severe stress in Latin American population.

Variable	Severe stress or >		cPR (IC 95%) *p*-value	
	No *n* (%)	Yes *n* (%)	Bivariate	Multivariate
World War III				
I am not afraid	1,597 (92.8)	124 (7.2)	Comparison category	Comparison category
Yes, I am afraid	749 (82.8)	156 (17.2)	2.39 (1.92–2.99) <0.001	2.05 (1.63–2.57) <0.001
Country				
Others	156 (94.6)	9 (5.4)	Comparison category	Comparison category
Colombia	506 (86.6)	78 (13.4)	2.45 (1.26–4.78) 0.009	2.10 (1.08–4.07) 0.028
Paraguay	350 (86.2)	56 (13.8)	2.53 (1.28–4.99) 0.008	2.55 (1.30–4.99) 0.007
Peru	363 (92.4)	30 (7.6)	1.40 (0.68–2.88) 0.362	1.53 (0.75–3.14) 0.246
Bolivia	296 (89.4)	35 (10.6)	1.94 (0.95–3.94) 0.067	1.91 (0.94–3.86) 0.073
Ecuador	200 (84.0)	38 (16.0)	2.93 (1.45–5.89) 0.003	2.53 (1.26–5.05) 0.009
Mexico	183 (89.7)	21 (10.3)	1.89 (0.89–4.01) 0.099	1.79 (0.85–3.80) 0.125
Panama	167 (95.4)	8 (4.6)	0.84 (0.33–2.12) 0.709	0.79 (0.31–1.98) 0.609
Brazil	125 (96.2)	5 (3.8)	0.71 (0.24–2.05) 0.522	1.02 (0.35–2.98) 0.968
Age (years)*	24 (21–29)	22 (20–25)	0.95 (0.93–0.97) <0.001	0.97 (0.95–0.98) 0.001
Sex				
Woman	1,394 (86.9)	211 (13.1)	Comparison category	Comparison category
Man	952 (93.2)	69 (6.8)	0.51 (0.40–0.67) <0.001	0.61 (0.47–0.79) <0.001
Education				
Technician or <	246 (91.1)	24 (8.9)	Comparison category	Comparison category
University students	2,100 (89.1)	256 (10.9)	1.22 (0.82–1.82) 0.324	1.09 (0.74–1.60) 0.676

Crude prevalence ratios (cPR), adjusted prevalence ratios (aPR), 95% confidence intervals (95%CI) and value of ps were obtained with generalized linear models (Poisson family, log link function and models for robust variances). *Variable taken as quantitative; median (interquartile range) is shown in the descriptive part.

## Discussion

The association between the perceived fear of the possible outbreak of a world war and the anxiety, depression and stress in severe or very severe degrees is confirmed, as well as in previous conflicts it has also been shown that mental health is greatly affected. There is a greater probability of mental health disorders in the displaced population, as they have a greater need for mental health care services in territories affected by conflict (45). A study of Vietnam War veterans showed that the prevalence of post-traumatic stress disorder was 5.4%, that of depression was 8.3 and 5.4% of veterans had suicidal thoughts (46). Similarly, Gulf War veterans developed post-traumatic stress disorder, depression, and multiple chronic illnesses (47). Another study of British personnel who participated in the Iraq and Afghanistan conflicts showed that 6.2% had probable post-traumatic stress disorder and 21.9% had common mental disorders (48). Also, in the armed conflict in Colombia, the affected population developed emotional distress, depressive symptoms, anxiety and stress (49). As can be observed, most of the researchers inquire about those directly affected, but there are few that evaluate this in the general population, which is necessary, especially since we are still in the context of the pandemic.

As a secondary result, it is reported that the levels of anxiety, depression or severe stress were important in some countries, especially in the case of anxiety, reaching levels between 12 and16% in most of them. It is important to emphasize that these countries were severely affected by COVID-19, triggering damage to mental health. For example, one study revealed that in Latin America more mental health symptoms such as post-traumatic stress disorder, anxiety and depression were reported to be related to COVID-19 (50). Another study showed that Latin America had a prevalence rate of depression and anxiety of 32% (51). Also, a meta-analysis showed that the prevalence of anxiety and depression was 35% (52). Therefore, it is important that each country measures the affectation of its population in mental health issues, since each area had a different affectation than elsewhere, and this could be accentuated in some specific subpopulations.

The population with the highest frequency of the three pathologies was the female population. This has been demonstrated in a study from the University of Maryland where they found the prevalence of depressive and anxiety symptoms in Latin American and Caribbean countries, in which it was shown that the female gender and non-binary gender people had more anxious and depressive symptoms, compared with men during the COVID-19 pandemic (53); In addition, in European countries there is also evidence that women have low emotional stability, which could be a reason for them to experience more symptoms of neuroticism (54). Although no clear explanation has been found for this, it may be due to ovarian hormonal variations and the decrease in estrogen levels in women, which is why they are more prone to present these types of disorders (55). This is a clear example of a sub-population that is more affected than another, which should serve for the authorities to look for this in a more individualized way, which will then serve to generate specific programs for the improvement of mental health.

Finally, these three pathologies occurred to a lesser extent among those who were older, as demonstrated in different studies where it is observed that the young population is the one that presents stress, anxiety and depression more frequently (56, 57). This is since it is the population with more time exposed to technology and information from social networks that trigger the presence of greater concern for their future in relation to the consequences in the economic and environmental sphere that these problems can generate (58, 59). This should be corroborated, and we should see what are the other causes that generate that the youngest are the most affected by these stimuli of war and the possible concern for what is generated by it.

It is recognized that there is a limitation in terms of 100% extrapolation of the results obtained in this study due to the specific context of the Russia-Ukraine war and the possible mental health disturbances associated with this conflict. It is important to emphasize that the respondents from the 13 selected countries were not chosen randomly, but rather based on the possibility of accessing the target population and on the connections and prior knowledge of the researchers in those countries. Although an effort was made to ensure the representativeness of the sample to the extent possible, it is recognized that this may present certain limitations.

Although there is limited evidence that mental health affects the SDG 3 (60–64), the present study highlights this relationship as it is a dynamic aspect since the negative impact that had been generated by COVID-19 is continued by all the effect generated by the concerns related to the escalation of the war worldwide. Efforts to achieve the Sustainable Development Goals are multidimensional because the contribution of this study is to show the importance of mental health in sustainability, even beyond just SDG 3.

We had the limitation of not being able to reach the older or rural population, mainly due to the type of design, whereby conducting a virtual survey we had more reach to the populations that have more use of technology and that are in cities with greater coverage of telephone and communication networks. In addition, our results cannot evaluate causality, so it is not possible to know whether it is the fear of a third world war that generates an increase in pathologies of the mental sphere or vice versa. In addition, by relying on surveys we had the bias of reaching only the accessible population that wanted to answer the surveys. However, despite all these limitations, we had access to more than two thousand people in Latin American in the end of the pandemic, which can generate a first report of this population, which was so hard hit by the pandemic, where Peru, Brazil and Argentina were three of the countries with the highest mortality globally (65). This generates more hypotheses and supports further research on mental health issues.

## Conclusion

Based on the above findings, it is concluded that a strong association was found between fear of a possible world war and having severe or very severe levels of anxiety, depression or stress in the Latin American population surveyed during the first weeks of the Russia-Ukraine conflict. It is important to consider that sustainability can be achieved to the extent that the SDGs can be met. In the case of SDG 3, and specifically in target 3.4, a great deal of effort will be required from governments since, as a war is ongoing, mental health was negatively affected, and if we add the COVID-19 pandemic, the population will continue to be affected, with all the social and economic impact that this implies.

It is important to keep in mind that this exploratory study represents a first approach to the association between severe anxiety, depression and stress in the Latin American population in the context of the aforementioned conflict. In order to obtain more solid and generalizable conclusions, future research will be necessary to include a more representative and random sample of the Latin American region, taking into consideration the diversity of contexts and socio-political situations.

## Data availability statement

The raw data supporting the conclusions of this article will be made available by the authors, without undue reservation.

## Ethics statement

The studies involving human participants were reviewed and approved by the committee gave its approval (Institutional Research Ethics Committee of the Norbert Wiener University, file 1,648–2022). The patients/participants provided their written informed consent to participate in this study.

## Author contributions

CM, SC-E, JC-L, MP, VP-F, JA, MV-E, and VS-A: conceptualization, methodology, software, validation, formal analysis, investigation, resources, and data curation. CM, SC-E, JC-L, MP, VP-F, JA, MV-E, VS-A, SD-A-A, AA-R, and JY: writing—original draft preparation, writing—review and editing, and visualization. All authors contributed to the article and approved the submitted version.

## Conflict of interest

The authors declare that the research was conducted without any commercial or financial relationships that could be construed as a potential conflict of interest.

## Publisher’s note

All claims expressed in this article are solely those of the authors and do not necessarily represent those of their affiliated organizations, or those of the publisher, the editors and the reviewers. Any product that may be evaluated in this article, or claim that may be made by its manufacturer, is not guaranteed or endorsed by the publisher.

## References

[1] United Nations. (2022). Sustainable development goals. Available at: https://sdgs.un.org/es/goals (Accessed March 03, 2023).

[2] United Nations. (2023). Good health and well-being. Available at: https://www.globalgoals.org/goals/3-good-health-and-well-being (Accessed March 03, 2023).

[3] Stucchi-Portocarrero S. La Primera Guerra Mundial y su impacto en la psiquiatría. Rev Neuropsiquiatr. (2014) 77:139–43. doi: 10.20453/rnp.v77i3.2028

[4] Del-Aguila-Arcentales S Alvarez-Risco A Villalobos-Alvarez D Carhuapoma-Yance M Yáñez JA. COVID-19, mental health and its relationship with workplace accidents. Int J Ment Health Promot. (2022) 24:503–9. doi: 10.32604/ijmhp.2022.020513

[5] GavinBLyneJMcNicholasF. Mental health and the COVID-19 pandemic. Ir J Psychol Med. (2020) 37:156–8. doi: 10.1017/ipm.2020.7232475368

[6] Pfefferbaum B North CS. Mental health and the COVID-19 pandemic. N Engl J Med. (2020) 383:510–2. doi: 10.1056/NEJMp2008017 32283003

[7] Renaud-Charest O Lui LMW Eskander S Ceban F Ho R Di Vincenzo JD. Onset and frequency of depression in post-COVID-19 syndrome: a systematic review. J Psychiatr Res. (2021) 144:129–37. doi: 10.1016/j.jpsychires.2021.09.054, PMID: 34619491 PMC8482840

[8] Zhang SX Miller SO Wen X Yin A Chen BZ Delios A. Meta-analytic evidence of depression and anxiety in Eastern Europe during the COVID-19 pandemic. Eur J Psychotraumatol. (2022) 13:2000132. doi: 10.1080/20008198.2021.2000132, PMID: 35186214 PMC8856103

[9] Ettman CK Abdalla SM Cohen GH Sampson L Vivier PM Galea S. Prevalence of depression symptoms in US adults before and during the COVID-19 pandemic. JAMA Netw Open. (2020) 3:e2019686. doi: 10.1001/jamanetworkopen.2020.19686 32876685 PMC7489837

[10] Chen X Zhang SX Jahanshahi AA Alvarez-Risco A Dai H Li J. Belief in a COVID-19 conspiracy theory as a predictor of mental health and well-being of health Care Workers in Ecuador: cross-sectional survey study. JMIR Public Health Surveill. (2020) 6:e20737. doi: 10.2196/20737, PMID: 32658859 PMC7375774

[11] Peteet JR. COVID-19 anxiety. J Relig Health. (2020) 59:2203–4. doi: 10.1007/s10943-020-01041-4, PMID: 32415426 PMC7227179

[12] Chernyak BV Popova EN Prikhodko AS Grebenchikov OA Zinovkina LA Zinovkin RA. COVID-19 and oxidative stress. Biochem Mosc. (2020) 85:1543–53. doi: 10.1134/S0006297920120068, PMID: 33705292 PMC7768996

[13] Janiri D Carfì A Kotzalidis GD Bernabei R Landi F Sani G. Posttraumatic stress disorder in patients after severe COVID-19 infection. JAMA Psychiat. (2021) 78:567–9. doi: 10.1001/jamapsychiatry.2021.0109, PMID: 33599709 PMC7893542

[14] Yan J Kim S Zhang SX Foo M-D Alvarez-Risco A Del-Aguila-Arcentales S. Hospitality workers’ COVID-19 risk perception and depression: a contingent model based on transactional theory of stress model. Int J Hosp Manag. (2021) 95:102935. doi: 10.1016/j.ijhm.2021.102935, PMID: 36540684 PMC9756832

[15] Belcher O Bigger P Neimark B Kennelly C. Hidden carbon costs of the “everywhere war”: logistics, geopolitical ecology, and the carbon boot-print of the US military. Trans Inst Br Geogr. (2020) 45:65–80. doi: 10.1111/tran.12319

[16] Branch A. From disaster to devastation: drought as war in northern Uganda. Disasters. (2018) 42:S306–27. doi: 10.1111/disa.12303, PMID: 30080269

[17] Greenstone M He G Li S Zou EY. China’s war on pollution: evidence from the first 5 years. Rev Environ Econ Policy. (2021) 15:281–99. doi: 10.1086/715550

[18] Certini G Scalenghe R Woods WI. The impact of warfare on the soil environment. Earth Sci Rev. (2013) 127:1–15. doi: 10.1016/j.earscirev.2013.08.009

[19] Radziemska M Bęś A Gusiatin ZM Cerdà A Mazur Z Jeznach J. The combined effect of phytostabilization and different amendments on remediation of soils from post-military areas. Sci Total Environ. (2019) 688:37–45. doi: 10.1016/j.scitotenv.2019.06.190, PMID: 31228768

[20] Venus M Puntarić D Gvozdić V Vidosavljević D Bijelić L Puntarić A. Determinations of uranium concentrations in soil, water, vegetables and biological samples from inhabitants of war affected areas in eastern Croatia (ICP-MS method). J Environ Radioact. (2019) 203:147–53. doi: 10.1016/j.jenvrad.2019.03.004, PMID: 30913484

[21] Li X-Y Li X Fan Z Mi L Kandakji T Song Z. Civil war hinders crop production and threatens food security in Syria. Nature Food. (2022) 3:38–46. doi: 10.1038/s43016-021-00432-4, PMID: 37118486

[22] National Geographic España. (2023). “Segunda Guerra Mundial.” Available at: https://historia.nationalgeographic.com.es/temas/segunda-guerra-mundial (Accessed March 03, 2023).

[23] Chaaya C Thambi VD Sabuncu Ö Abedi R Osman AOA Uwishema O. Ukraine – Russia crisis and its impacts on the mental health of Ukrainian young people during the COVID-19 pandemic. Ann Med Surg. (2022) 79:104033. doi: 10.1016/j.amsu.2022.104033, PMID: 35765517 PMC9221679

[24] Hellegers P. Food security vulnerability due to trade dependencies on Russia and Ukraine. Food Secur. (2022) 14:1503–10. doi: 10.1007/s12571-022-01306-8, PMID: 35891962 PMC9304541

[25] Uwishema O Sujanamulk B Abbass M Fawaz R Javed A Aboudib K. Russia-Ukraine conflict and COVID-19: a double burden for Ukraine’s healthcare system and a concern for global citizens. Postgrad Med J. (2022) 98:569–71. doi: 10.1136/postgradmedj-2022-141895 35654572 PMC9340026

[26] De Coninck D. The refugee paradox during wartime in Europe: how Ukrainian and afghan refugees are (not) alike. Int Migr Rev. (2022) 57:578–86. doi: 10.1177/01979183221116874

[27] Mearsheimer J. (2022). “The causes and consequences of the Ukraine war - CIRSD.” Available at: https://www.cirsd.org/en/horizons/horizons-summer-2022-issue-no.21/the-causes-and-consequences-of-the-ukraine-war (Accessed March 03, 2023).

[28] Sloss DL Dickinson LA. The Russia-Ukraine war and the seeds of a new Liberal Plurilateral order. Am J Int Law. (2022) 116:798–809. doi: 10.1017/ajil.2022.55

[29] Ali S. Mahmud. (2022). “Introduction: a new cold war? The US-China-Russia strategic triangle.” In The US-China-Russia triangle: an evolving historiography, edited by Ali S. Mahmud, 1–40. Cham: Springer International Publishing.

[30] Ali SM. The triangle in world war and its wake In: Mahmud Ali S, editor. The US-China-Russia triangle: an evolving historiography. Cham: Springer International Publishing (2022). 131–89.

[31] Batyuk VI Morozov YV. Politics and strategy of the United States, the Russian Federation, and China in the Middle East. Her Russ Acad Sci. (2022) 92:S321–30. doi: 10.1134/S1019331622100021

[32] BBC. (2023). Ukraine in maps: tracking the war with Russia. Available at: https://www.bbc.com/news/world-europe-60506682 (Accessed March 03, 2023).

[33] Choudhary OP Saied ARA Priyanka RK Ali RK Maulud SQ. Russo-Ukrainian war: an unexpected event during the COVID-19 pandemic. Travel Med Infect Dis. (2022) 48:102346. doi: 10.1016/j.tmaid.2022.102346, PMID: 35487342 PMC9042412

[34] Dhawan M Choudhary OP Choudhary P Saied ARA. Russo-Ukrainian war amid the COVID-19 pandemic: global impact and containment strategy. Int J Surg. (2022) 102:106675. doi: 10.1016/j.ijsu.2022.106675, PMID: 35569761 PMC9098802

[35] Aymerich C Pedruzo B Pérez JL Laborda M Herrero J Blanco J. COVID-19 pandemic effects on health worker’s mental health: systematic review and meta-analysis. Eur Psychiatry. (2022) 65:e10. doi: 10.1192/j.eurpsy.2022.1, PMID: 35060458 PMC8828390

[36] Dragioti E Li H Tsitsas G Lee KH Choi J Kim J. A large-scale meta-analytic atlas of mental health problems prevalence during the COVID-19 early pandemic. J Med Virol. (2022) 94:1935–49. doi: 10.1002/jmv.27549, PMID: 34958144 PMC9015528

[37] Schwalb A Armyra E Méndez-Aranda M Ugarte-Gil C. COVID-19 in Latin America and the Caribbean: two years of the pandemic. J Intern Med. (2022) 292:409–27. doi: 10.1111/joim.13499, PMID: 35411985 PMC9115176

[38] Alarcón-Braga EA Hernandez-Bustamante EA Salazar-Valdivia FE Valdez-Cornejo VA Mosquera-Rojas MD Ulloque-Badaracco JR. Acceptance towards COVID-19 vaccination in Latin America and the Caribbean: a systematic review and meta-analysis. Travel Med Infect Dis. (2022) 49:102369. doi: 10.1016/j.tmaid.2022.102369, PMID: 35680058 PMC9169427

[39] Rosiello DF Anwar S Yufika A Adam RY Ismaeil MIH Ismail AY. Acceptance of COVID-19 vaccination at different hypothetical efficacy and safety levels in ten countries in Asia, Africa, and South America. Narra J. (2021) 1:1–16. doi: 10.52225/narra.v1i3.55 PMC10914086 38450212

[40] Caglevic C Rolfo C Gil-Bazo I Cardona A Sapunar J Hirsch FR. The armed conflict and the impact on patients with Cancer in Ukraine: urgent considerations. JCO Global Oncol. (2022) 8:e2200123. doi: 10.1200/GO.22.00123, PMID: 35994695 PMC9470147

[41] Vivalya BM Nzanzu MM Vagheni GM Kitoko B Vutegha JM Kalume AK. Developing mental health services during and in the aftermath of the Ebola virus disease outbreak in armed conflict settings: a scoping review. Glob Health. (2022) 18:71. doi: 10.1186/s12992-022-00862-0, PMID: 35836283 PMC9281256

[42] Moghanibashi-Mansourieh A. Assessing the anxiety level of Iranian general population during COVID-19 outbreak. Asian J Psychiatr. (2020) 51:102076. doi: 10.1016/j.ajp.2020.102076, PMID: 32334409 PMC7165107

[43] Ozamiz-Etxebarria N Mondragon NI Bueno-Notivol J Pérez-Moreno M Santabárbara J. Prevalence of anxiety, depression, and stress among teachers during the COVID-19 pandemic: a rapid systematic review with Meta-analysis. Brain Sci. (2021) 11:1172. doi: 10.3390/brainsci11091172, PMID: 34573192 PMC8468121

[44] Román F Santibáñez P Vinet EV. Uso de las Escalas de Depresión Ansiedad Estrés (DASS-21) como instrumento de tamizaje en jóvenes con problemas clínicos. Acta de investigación psicológica. (2016) 6:2325–36. doi: 10.1016/S2007-4719(16)30053-9

[45] León-Giraldo S Casas G Cuervo-Sánchez JS García T González-Uribe C Moreno-Serra R. Trastornos de salud mental en población desplazada por el conflicto en Colombia: análisis comparado frente a la Encuesta Nacional de salud mental 2015. Revista Colombiana de Psiquiatría. (2021) 52:121–9. doi: 10.1016/j.rcp.2021.04.012 37453820

[46] Boscarino JA Adams RE Urosevich TG Hoffman SN Lester Kirchner H Boscarino JJ. Mental health impact of homecoming experience among 1730 formerly deployed veterans from the Vietnam war to current conflicts: results from the Veterans’ health study. J Nerv Ment Dis. (2018) 206:757–64. doi: 10.1097/NMD.0000000000000879, PMID: 30273271 PMC6171364

[47] Blakey SM Halverson TF Evans MK Patel TA Hair LP Meyer EC. Experiential avoidance is associated with medical and mental health diagnoses in a national sample of deployed gulf war veterans. J Psychiatr Res. (2021) 142:17–24. doi: 10.1016/j.jpsychires.2021.07.033, PMID: 34314990 PMC8429252

[48] Stevelink SAM Jones M Hull L Pernet D MacCrimmon S Goodwin L. Mental health outcomes at the end of the British involvement in the Iraq and Afghanistan conflicts: a cohort study. Br J Psychiatry. (2018) 213:690–7. doi: 10.1192/bjp.2018.175, PMID: 30295216 PMC6429255

[49] Campo-Arias A Sanabria AR Ospino A Guerra VM Caamaño BH. Multiple-victimisation due to armed conflict and emotional distress in the state of Magdalena, Colombia. Revista Colombiana de Psiquiatría. (2017) 46:147–53. doi: 10.1016/j.rcpeng.2016.06.002, PMID: 28728798

[50] Olff M Primasari I Qing Y Coimbra BM Hovnanyan A Grace E. Mental health responses to COVID-19 around the world. Eur J Psychotraumatol. (2021) 12:1929754. doi: 10.1080/20008198.2021.1929754, PMID: 34262666 PMC8253206

[51] Zhang SX Chen J. Scientific evidence on mental health in key regions under the COVID-19 pandemic – meta-analytical evidence from Africa, Asia, China, Eastern Europe, Latin America, South Asia, Southeast Asia, and Spain. Eur J Psychotraumatol. (2021) 12:2001192. doi: 10.1080/20008198.2021.2001192, PMID: 34900123 PMC8654399

[52] Zhang SX Batra K Wen X Liu T Dong RK Yin A. Mental disorder symptoms during the COVID-19 pandemic in Latin America – a systematic review and meta-analysis. Epidemiol Psychiatr Sci. (2022) 31:e23. doi: 10.1017/S2045796021000767, PMID: 35438066 PMC9069590

[53] Herrera-Añazco P Urrunaga-Pastor D Benites-Zapata VA Bendezu-Quispe G Toro-Huamanchumo CJ Hernandez AV. Gender differences in depressive and anxiety symptoms during the first stage of the COVID-19 pandemic: a cross-sectional study in Latin America and the Caribbean. Front Psych. (2022) 13:7034. doi: 10.3389/fpsyt.2022.727034 PMC8968114 35370810

[54] Brito-Costa S Jonason PK Tosi M Antunes R Silva S Castro F. Opinions and options about COVID-19: personality correlates and sex differences in two European countries. PLoS One. (2022) 17:e0268193. doi: 10.1371/journal.pone.0268193, PMID: 35657914 PMC9165842

[55] Albert PR. Why is depression more prevalent in women? J Psychiatry Neurosci. (2015) 40:219–21. doi: 10.1503/jpn.150205, PMID: 26107348 PMC4478054

[56] Salari N Hosseinian-Far A Jalali R Vaisi-Raygani A Rasoulpoor S Mohammadi M. Prevalence of stress, anxiety, depression among the general population during the COVID-19 pandemic: a systematic review and meta-analysis. Glob Health. (2020) 16:57. doi: 10.1186/s12992-020-00589-w, PMID: 32631403 PMC7338126

[57] Ahmed MZ Ahmed O Aibao Z Hanbin S Siyu L Ahmad A. Epidemic of COVID-19 in China and associated psychological problems. Asian J Psychiatr. (2020) 51:102092. doi: 10.1016/j.ajp.2020.102092, PMID: 32315963 PMC7194662

[58] Huang Y Zhao N. Generalized anxiety disorder, depressive symptoms and sleep quality during COVID-19 outbreak in China: a web-based cross-sectional survey. Psychiatry Res. (2020) 288:112954. doi: 10.1016/j.psychres.2020.112954, PMID: 32325383 PMC7152913

[59] Schneider-Mayerson M Leong KL. Eco-reproductive concerns in the age of climate change. Clim Chang. (2020) 163:1007–23. doi: 10.1007/s10584-020-02923-y

[60] Herrman H. Sustainable development goals and the mental health of resettled refugee women: a role for international organizations. Front Psych. (2019) 10:608. doi: 10.3389/fpsyt.2019.00608, PMID: 31543834 PMC6728808

[61] Mills C. From ‘invisible problem’ to global priority: the inclusion of mental health in the sustainable development goals. Dev Chang. (2018) 49:843–66. doi: 10.1111/dech.12397

[62] Stein DJ Benjet C Gureje O Lund C Scott KM Poznyak V. Integrating mental health with other non-communicable diseases. BMJ. (2019) 364:l295. doi: 10.1136/bmj.l295, PMID: 30692081 PMC6348425

[63] Thornicroft G Votruba N. Sustainable development goals and mental health. Vertex. (2018) 29:300–3. 30785971

[64] WHO. (2019). “WHO global meeting to accelerate progress on SDG target 3.4 on noncommunicable diseases and mental health.” Available at: https://apps.who.int/iris/handle/10665/334384 (Accessed March 03, 2023). 10.26719/2021.27.5.524 34080682

[65] WHO. (2022). “Coronavirus dashboard.” Available at: https://covid19.who.int (Accessed March 03, 2023).

